# Factors influencing visitors’ use of augmented reality technology in museum guided tours

**DOI:** 10.1371/journal.pone.0332688

**Published:** 2025-10-07

**Authors:** Ying He, Wenyue Wang

**Affiliations:** 1 School of cultural industries and tourism, Xiamen University of Technology, Xiamen, China; 2 School of Communication, Liaoning Communication University, Shenyang, China; Politecnico di Torino, ITALY

## Abstract

The emergence of Augmented Reality (AR) technology has provided innovative ideas for museum tourism services, creating a unique tour experience for visitors. However, there is little research known for the user’s behavior of using AR technology in the context of interactive museum guided tour services. This study aimed to explore the factors that drive tourists to use AR guided tours in museums. Based on the Stimuli-Organism-Response framework and the Information System Success Model, this study attempted to construct a model of visitor acceptance of AR guided museum tour for explaining and predicting visitors’ behaviors of AR guided museum tours. Structural equation modeling was used in this study to analyze the data from 324 survey questionnaires. The results showed that individuation and interactivity positively affected satisfaction and perceived enjoyment, authenticity and aesthetics positively affected perceived enjoyment, and perceived enjoyment positively affected satisfaction. In addition, perceived enjoyment and satisfaction positively affected the intention to use AR guided tours. The study has revealed the factors affecting the intention to use AR guided tours, extended the application of the Stimuli-Organism-Response framework and the Information System Success Model, and enriched the theories of museum user behavior. This study can provide valuable references for museum managers, tourism marketers and AR technology developers to enhance the AR tour experience of museum visitors and promote the healthy development of museum tourism.

## Introduction

With the improvement of economic level and people’s spiritual and cultural needs, visiting museums has become one of the cultural tourism and leisure activities. At the same time, museums have become a place of informal education due to the transformation of social structure. As a carrier of regional culture, museums have significant functions such as collection, exhibition, scientific research and education, providing an important spatial field for the public to understand local history and culture, to absorb cultural knowledge and enjoy cultural tourism. Nowadays, tourists also hope to acquire knowledge and have a relaxing and pleasant experience during their visits to museums [1]. New digital technologies play an increasingly important role in museums and gradually become inseparable from museums, with intelligent technologies including artificial intelligence (AI), augmented reality (AR), virtual reality (VR), and the Internet of Things [2]. Museum tours with digital technologies have changed the way information and knowledge are disseminated, creating a unique experience for visitors [3]. The attempts to apply the 360-degree panoramic projection began as early as in the 19th century to present the media content in a novel manner to create a new visitor experience [4], this further highlights that visiting museums is pleasant and enjoyable [5]. Therefore, it has become a crucial part of the intelligent construction of museums to continuously improve tourists’ smart tourism experience.

AR overlays digital content on the real environment and objects, offering a multidimensional context for users [6]. It can be combined with smart glasses, smartphones or VR headsets to provide users with rich information [7]. Augmented reality is regarded as one of the most promising digital technologies that can bring rich experiences to visitors [8]. For instance, Grammatikopoulou and Grammalidis [9] proposed a funded tour guide tool based on augmented reality technology to facilitate museum Tours and interactions for visitors. The museum Augmented Reality technology guided tour is based on Augmented Reality (AR) technology. Through mobile devices or dedicated hardware devices, the real exhibits in the museum are integrated with virtual data, enabling visitors to perceive the information of the exhibits and bringing users a unique visiting experience. Under the background of the integration of culture and tourism, museums and other attractions are facing the pressure of attracting tourists to visit and experience them [10]. At the same time, previous research [11] pointed out that tourists know little about the destination or the object of the visit, which prompts AR to bring new market opportunities in the tourism field, and user satisfaction can be greatly enhanced through advanced virtual digital technology. New immersive technologies such as AR are being applied to cultural attractions such as museums, creating a unique visitor experience through the integration of real and digital content [12]. It can enhance visitors’ learning of the knowledge of the architecture or collections [13]. Technological advances have influenced museum-related research, including the conservation, exhibition, guided tours and education of museum collections. Some studies have been conducted from different perspectives, such as AR learning in museum experiences [14,15], as well as AR and VR experiences in museums [16,17]. For example, the application of augmented reality technology to improve children’s cultural museums can enable children to learn actively and proactively in digital museums [18]. It can be said that the adoption of augmented reality technology in museums has become one of the important strategies to enhance the visiting experience of museum visitors [19].

Previous research on augmented reality technology in museums has mainly focused on exploring digital modeling and AR applications of the combination of digital technology and museum space [20], as well as environment generation and dynamic tracking [21], and digital interaction and digital management of exhibits [22]. Besides, Previous studies [23] have focused on the visitor experience in museums, including the use of audio guided tours [24], smartphones [25] and other technologies to enrich the user experience. With the application of AR in museums and other cultural tourism at-tractions, it has also gradually attracted the attention of academics how visitors’ AR experience affects their behaviors [26]. However, except for only a few studies [27,28] that have investigated the impact of visitors’ AR experience on their attitudes towards and loyalty to AR, the study of how AR guided tours drive users’ technology behaviors is still a new area that has not been systematically explored [26]. Furthermore, some museums have difficulty in determining effective methods for digital technology exhibitions [29], and due to the poor performance of digital technology companies in attracting museum visitors, there is a disconnect between the digital technologies applied by museums and users’ digital lives [30]. Arguably, while previous studies have focused on the potential of AR to enhance visitor experience in such domains as museums and tourism [11,31,32], little is known about the user behavior of using AR in the context of interactive services [33], such as in the context of museum guided tours. Only a few studies have focused on the use of augmented reality technology in museum exhibits and the challenges it faces [34]. That is to say, it is extremely important for museums and the developers and designers of digital technologies to explore the variables that affect the acceptance degree of museum visitors to the digital technology service of museum AR guided Tours, as well as the relationships among these variables. Therefore, one of the objectives of this study is to focus on museum AR guided tours and explore the factors that influence tourists’ use of AR guided tours from the tourists’ perspective.

A number of theories have been developed to identify the factors that influence visitors’ intention to use new technologies [35]. One of these theories is the Information Systems Success Model (ISSM) [36], which has been widely used to explain users’ technology use behavior. The ISSM describes how users’ intention to use a technology is influenced by the information system, the quality of the service, the quality of the system and satisfaction. Previous studies [11,37] have demonstrated the AR technology has an impact on the tourism industry, including museums, and is one of the key drivers of tourism innovation. Therefore, exploring the interrelationships between AR technology and tourists’ behavioral intentions can facilitate a more in-depth understanding of tourists’ perceptions of AR guided tours in museums and users’ technology use behaviors, and promote the sustainable development of museum tourism. Therefore, one of the objectives of this study is to expand the ISSM and its application areas by exploring how the information quality, service quality and satisfaction of museum tourists with AR technology affects their intention to use AR technology, with the ISSM as a theoretical basis.

Of course, the user’s behavior of technology acceptance is complex with a psychological perception process, including the influence of external factors in the early stage, which triggers the user’s psychological perception and finally produces a series of subsequent behavioral intentions. This process may be consistent with the content of Stimuli-Organism-Response (SOR) framework [38]. SOR states that user behavior is generated by internal psychological responses or perceptions based on external stimuli. It provides a theoretical basis for researchers to explore consumer behavior [39]. Tourists’ use behavior of new technologies is an important topic in academia and has been a focus of attention in the tourism industry for many years [40]. The widespread popularization of museum AR guided tour technology requires a systematic study of the decision-making process of museum visitors in the acceptance and use of AR technology. Therefore, in order to gain a more systematic insight into the process of users’ acceptance and use of AR technology in museum tourism, the third objective of this study is to explore the applicability of the SOR framework in museum AR guided tours, and attempt to introduce the SOR framework into tourists’ AR technology behaviors, so as to enrich the research on the application of the SOR framework in museum tourism scenarios.

The integration of AR in museums can provide a unique visiting experience for visitors in terms of museum guidance and visualization [41]. In the context of digital museums, related research on visitors’ behavior and their museum information technology systems mainly focuses on comprehensive case studies for museums [42–44]. And most of them are qualitative studies, focusing on exploring concepts, exhibition and display proposals, as well as technological implementations, etc. [45,46], and lacking of research on specific contexts such as museum guided tours. At the same time, little is known about the study of user’s behavior of using AR in the context of interactive services [33]. Research on the application of AR in museums is still insufficient [1]. Conducting research on visitors’ usage behavior of augmented reality technology in museums is of great significance for predicting users’ experiences of using augmented reality in museum contexts [1]. However, what is the tourists’ level of acceptance of museum AR guided tours? What are the factors that influence the user’s behavior of using museum AR guided tours? Answering these questions is crucial to understand the requirements of future visitors and the digitalization construction of museums. Therefore, this study explored the factors affecting users’ intention to use AR guided tours in the context of museum AR guided tours from the perspective of tourists, using SOR as a theoretical framework and the ISSM as a theory. The study attempted to explore the influence of authenticity, individuation, aesthetics, and interactivity as stimuli, and perceived enjoyment and satisfaction as organisms, on the response of users’ intention to use AR guided tours. The results of this study can provide insights for museum tourism managers and AR developers to effectively enhance guided tour ser-vices, enhance museum visitors’ tour experiences, promote the preservation and promotion of cultural heritage in museums, and facilitate the construction of museum tourism and digitization. Overall, the contribution of this study is mainly manifested in the following aspects. Firstly, based on the SOR theory, a new conceptual model has been added to the existing literature by exploring the factors influencing the intention of museum visitors to use AR guided Tours. Secondly, this research has significant reference value for the management and design practice of museums and their related institutions. The subsequent structure of this paper includes: First, combined with previous studies, research hypotheses and conceptual models are proposed. Secondly, the research objects, tools and data analysis methods were expounded. Thirdly, the results were analyzed and discussed. Fourth, the theoretical and practical contributions, limitations and future development directions of this research were further clarified.

### Theoretical basics and research hypotheses

The SOR model originated from the field of environmental psychology and provides an ordered mechanism for understanding the complexity of user behavior [47]. It explains the influence of external stimuli on users’ internal cognitive states, emotional responses and emotions, thereby shaping subsequent behaviors, and is widely used in the analysis and research of users’ technical behaviors [48]. Considering that the SOR framework is an excellent evaluation tool for user behavior analysis and decision-making processes [49,50], this study will take the SOR theory as the research framework. In terms of the effectiveness or success of information systems, DeLone and McLean proposed a widely accepted success model of information systems in 1992. This model can be interpreted as follows: The system can be evaluated based on information, system and service quality, and these characteristics affect subsequent usage or usage intentions as well as user satisfaction. This model has been widely applied in the research on users’ intentions to adopt new technologies [51,52]. Considering that the information system success model is one of the most widely adopted frameworks for the study of user behavior in information technology [53]. Therefore, this study takes it as one of the theoretical models.This study fully takes into account that the applicability of the SOR framework in explaining users’ complex behaviors in different situations has been verified in previous rich studies [54]. Meanwhile, the flexibility of this theoretical framework and its ability to obtain various variables in different supporting theories [55], and the information system success model is of guiding significance for the evaluation of user behavior decisions in new technological systems. Moreover, in museum services, the quality of information and services is crucial for the success of museum Tours. These two variables are the key factors in the success model of the information system. Therefore, in the context of museum guided Tours, this study attempts to integrate the SOR framework and the successful model of the information system to explain and predict the behavior of museum visitors using AR guided Tours.

#### SOR.

SOR is a framework that is mainly used to explore the individual psychological response to the external stimulus and its subsequent behaviors [38]. The SOR frame-work includes three parts: stimuli-organism-response, in which the stimuli represent the stimuli that lead to the individual’s response; the organism represents the subject of the response, which refers to the individual’s inner activities or emotional state after being stimulated, including excitement, pleasure, etc.; the response represents the stimulus-induced reaction, which is the individual’s affinity or avoidance behavior triggered by the user’s psychological state after being externally stimulated. This framework explains the impact of external stimuli on users’ emotions and subsequent behaviors. Considering that this framework has been widely used in e-commerce [56], consumer shopping [57] and other fields, and its applicability has been verified, it may also be applicable to the scenario of museum AR guided tours. At the same time, it is considered that users’ mental processes and behaviors may be more complex in the museum context when they are facing the emerging technology of AR guided tours. However, the SOR framework provides a scientific and reasonable sequential mechanism for understanding the complexity of human behavior [47], and the theoretical model has high flexibility and expandability. For example, Kim, Lee [58] explored tourists’ virtual tourism behaviors and their intentions by using the extended SOR framework. In the context of augmented reality, the amount of information stimulus added to the user’s visual perception will affect the user’s technical usage experience and behavioral decisions, especially if it can supplement and enrich the existing information in the existing physical environment [59]. Just as Amorim, Guerreiro [60] pointed out, the technical experience of users in augmented reality can be attributed to the SOR model. Therefore, this study took SOR as the basic framework. In this study, authenticity, individuation, aesthetics and interactivity were taken as stimuli, perceived enjoyment and satisfaction as organisms, and intention to use the AR guided tour as response.

#### ISSM.

Delone and McLean [36] constructed and validated an Information System Success Model, a theory that is often widely used in studies of the users of technology systems. The model [36] states that information quality, system quality and service quality have an impact on user satisfaction and intention to use. The ISSM states that information quality is an important factor in the success of a technology system. In-formation quality is understood as the quality of the content and form produced by a technology system, and has been measured by scholars in terms of different dimensions such as comprehensibility, timeliness, reliability and accuracy [36,61,62]. Its positive effect on users’ intention to use technology has been confirmed [60]. It can also be interpreted as the extent to which users receive complete, accurate, authentic and timely information through information technology [63]. In the context of museum AR guided tours, the quality of content and service provided by AR guided tours should be of greater concern to visitors. Therefore, it is especially important to explore the content quality and service quality of museum AR guided tours in order to explain visitors’ behavioral intention. The theoretical framework of this research includes two factors in the success model of the information system, namely service quality and information quality. Considering museum AR guided tours as an assistive technology, the quality of the information presented should be authentic and individualized, as only in this way can it be understood and impressed by the visitors. True and reliable information [64] and personalized services [65] can enhance user satisfaction. Therefore, this study took authenticity and individuation as the content of information quality. In addition, as a service technology, the main purpose of museum AR guided tours is to provide users with a good tour service experience, but in the process of this service, users’ service experience may be affected by the presentation form and interaction form of the guided tour information of this technology. Therefore, this study took interactivity and aesthetics as the content of service quality.

#### Hypothesis development.

##### Authenticity:

Authenticity refers to the holistic nature of the museum AR guided tour experience [66], which focuses on how the guided tour experience or a particular guided tour process initiates visitors’ tour emotions when visiting a museum [67]. Dai et al. [68] subdivided authenticity into the objective authenticity, constructive authenticity and existential authenticity, which showed that authenticity had a positive impact on users’ satisfaction with heritage tourism. Wang [69] argued that in the context of museum AR guided tourism, authenticity should at least include the objective authenticity of the museum collection itself and the constructive authenticity of AR. Regardless of the type of authenticity, the current content of heritage authenticity and tourism authenticity is based on respecting cultural diversity and individual experiences [68]. Zhang, Yin [70] holds that authenticity includes objective truth, constructive truth, existential truth and postmodern truth. In this study, no matter which kind of authenticity it is, it has a positive impact on the satisfaction of tourists. Zerva [71] pointed out that authenticity can be considered as an individual’s experiential value, which influenced the individual’s behavioral decision-making process. Lee et al. [72] found that tourists’ perceptions of the authenticity of the objects they visit play an important role in predicting their behavior. In the context of cultural heritage tourism, authenticity has been shown to have a positive impact on satisfaction with the experience [73,74]. Engeset and Elvekrok [75] and Hede et al. [74] have confirmed the positive relationship between authenticity and satisfaction. Lu, et al. [76] considered authenticity as an important component of the original ecology, i.e., the original and authentic natural environment that has not been tapped by humans, and found that authenticity in camping tourism experience affected the perceived enjoyment that tourists get from their camping experience. Tussyadiah et al. [77], on the other hand, considered perceived presence as part of authenticity, and explored the positive effect of perceived presence on perceived enjoyment in virtual tourism. Therefore, this study proposed that:

H1: Authenticity has a positive effect on satisfaction.

H2: Authenticity has a positive effect on perceived enjoyment.

##### Individuation:

In this study, individuation was defined as the provision of real-time and accurate individualized services to users by the museum AR guided tour technology on the basis of mastering users’ preferences and needs, indicating that users can obtain relevant information that meets their needs [78]. A technical platform can mine and analyze users’ demands through their data information, provide personalized services for users, and thereby bring users rich experiences. Previous studies [79,80] have shown that individualized services from museum smart tourism technologies can meet the needs of tourists, maximize their tourism experience and increase their satisfaction with the smart tourism destination. Häubl and Trifts [81] demonstrated that individualized recommendation settings helped users to make high-quality decisions and significantly increased after-sales satisfaction. Tam and Ho [82] showed that individuation of mobile shopping platforms can improve consumers’ shopping experience, bring positive emotional responses, and increase their satisfaction. Meanwhile, Qian et al. [83] pointed out that individualized recommendations were the main driver of users’ emotional responses when they were shopping, because individualized settings could generate tai-lor-made pleasure for users. Liu et al. [84] also verified that the individuation of mobile shopping platforms could make users have pleasure. Pathak and Garfinkel [85] pointed out that personalized technological platforms can reduce users’ cognitive load, improve their decision-making level, and trigger positive emotional responses from users. In addition, Zhang, Leng and Liu [78] demonstrated that system individuation positively affected users’ perceived enjoyment. This study suggests that individuation can promote the generation of positive emotions during tourists’ AR guided tours, so the following two hypotheses were proposed:

H3: There is a significant positive relationship between individuation and satisfaction.

H4: There is a significant positive relationship between individuation and perceived enjoyment.

##### Aesthetics:

Aesthetics has been interpreted as the pleasing appearance of visual arts [86]. Han, Yoon et al. [26] interpreted visual attractiveness as aesthetic content, which satisfies tourists by providing them with an aesthetic experience through multi-sensory perceptions such as sight, sound and touch. The aesthetics of IT systems are mainly realized through visualized graphic symbols such as colors, shapes, and fonts [87]. The significance of aesthetics in AR applications is very significant because most of the current AR applications are based on the carriers of small mobile devices with small display sizes, which emphasizes the importance of aesthetics. Previous studies have demonstrated the relationship between aesthetics and technology system satisfaction. Jung, Lee [17] ‘s research found that esthetic experience is an important factor influencing users’ augmented reality experience of cultural heritage. Chung et al. [87] showed that the aesthetic experience of AR had an impact on visitors’ satisfaction with AR. Jung et al. [88] studied user acceptance of a location-based AR navigation system, and found that those aesthetic experiences gained through AR content positively affected users’ satisfaction. Tang et al. [89] verified that aesthetics in digital marketing positively affected customer satisfaction. The positive impact of aesthetics on perceived enjoyment has been confirmed in previous studies. For example, in the study of information retrieval on websites by Cyr et al. [90], it was noted that design aesthetics positively influenced users’ perceived enjoyment. Chung et al. [87] demonstrated the positive impact of aesthetics on visitors’ AR satisfaction and perceived enjoyment with the use of Korean cultural heritage as an example. Museums can offer visitors relaxation and aesthetic experiences [5]. As exhibitions in museums, they are not only interactive but also concrete and emotional, triggering aesthetic experiences [91], and aesthetic factors play an important role in promoting the visiting behavior of museum users [92]. Therefore, In this study, we hypothesized that museum AR applications that possess aesthetic qualities and provide users with an aesthetic experience are likely to satisfy users and enable them to have positive emotional responses. That is, we hypothesized that the aesthetic experience of museum AR guided tours will affect visitors’ satisfaction and perceived enjoyment. Therefore, we proposed the following:

H5: There is a significant positive relationship between aesthetics and satisfaction.

H6: There is a significant positive correlation between aesthetics and perceived enjoyment.

##### Interactivity:

Yang and Shen [93] interpreted interactivity as modal interaction, which allows users to easily interact and engage with information content. Interactivity promotes feedback from travelers, which is related to how visitors react to AR technology in museums. Wangming Hu and Hyunsuk Han [94] have confirmed that museums constructed using augmented reality technology can enhance the interaction effect between young and elderly users. Interactivity can bring pleasure to the user by triggering a sense of participation and interaction in a virtual environment [28]. The interactivity of virtual reality can enable users to perceive entertainment by triggering their participation experience [95]. Those virtual tours with dynamic displays and interactive features can connect visitors to the immersion in virtual environments [96], inducing perceived enjoyment of visitors. Nguyen et al. [97] suggested that interactivity is an important driver of perceived enjoyment for tourists. Both Novak et al. [98] and Chi et al. [99] confirmed that the higher the interactivity, the higher the user’s perceived enjoyment. Gu et al. [100] demonstrated that the interactivity feature of live shopping can directly enhance consumers’ perceived enjoyment experience. Meanwhile, in social media services, when users perceive higher interactivity, they tend to adopt the service, which generates purchasing behavior, comments and feedback [2], and will make users feel satisfied with the service. In addition, Jianguo [101] confirmed the positive effect of interactivity on satisfaction in his study on the experience of smart tourism technology in museums. Interactivity, as an important feature of augmented reality, it is necessary to incorporate it into the stimulating factors as a consideration for influencing users’ intention to use the technology [97]. Museum exhibitions, as an interactive experience [91], have a certain impact on users’ perception and behavior after integrating augmented reality technology. Therefore, the hypotheses of this study were proposed as follows:

H7: Interactivity has a positive effect on satisfaction.

H8: Interactivity has a positive effect on perceived enjoyment.

##### Perceived enjoyment:

Perceived enjoyment refers to the pleasurable feelings obtained from various experiences [102]. In the context of tourism, the key to the success or failure of a tourism experience is perceived experiential value [87], and perceived enjoyment is a key com-ponent of emotional experiential value [103], which affects the positive emotion of user satisfaction. It affects the positive emotions of user satisfaction and is full of the degree of happiness that users feel when using the technical system. Verhagen et al. [104] found that users’ perceived enjoyment experience had a positive impact on satisfaction. Nguyen, Le [97] It also confirmed that the pleasure perceived by tourists has a positive impact on satisfaction. Hyowon, Jungkun [92] Research in the context of art museums shows the positive impact of hedonism on the satisfaction of museum visits among millennials. Jung et al. [88] demonstrated the significant effect of entertainment experience on satisfaction in a study of an AR system. Meanwhile, previous studies [105] have confirmed that the entertainment perceived by users can play a role in virtual Tours, thereby triggering tourists’ intention to visit. Previous research [106] found that perceived enjoyment was positively related to users’ intention to use a website. Leveau and Camus [107] showed that the entertainment users experienced during VR marketing experiences positively shaped users’ intention to visit consumer tourist destinations. Amorim, Guerreiro [60] found that in the AR retail scenario, entertainment has a positive impact on users’ purchase intentions. In addition, Wojciechowski and Cellary [108] stated that perceived playfulness had a significant effect on learners’ behavioral intention in a study of VR learning. Zhang et al. [78] pointed out that the subjective emotions generated during the shopping process would increase the consumers’ active participation in the mobile store, which further enhanced the consumers’ impulsive purchase intention, i.e., the influence on the consumers’ impulsive purchase intention mainly came from the positive emotions, such as perceived enjoyment. Similarly, In Li, Tu [1] ‘s research on museum AR technology visiting behavior based on the technology acceptance model, it was found that perceptual entertainment can positively influence users’ intention to use AR technology. Wu, Jiang [43] pointed out that the use of AR technology in clothing museums would generate more positive emotions such as hedonism and stimulate users’ intention to use the technology. Therefore, in the context of museum AR guided tours, visitors’ perceived enjoyment may also have a positive impact on their satisfaction and behavioral intention. Therefore, it was proposed that:

H9: Perceived enjoyment has a positive effect on satisfaction.

H10: Perceived enjoyment has a positive effect on intention to use AR guided tours.

##### Satisfaction:

The psychological state of users will affect their use of augmented reality technology [109], and satisfaction is one of the important psychological perceptions of users. Jeong and Shin [110] interpreted satisfaction as a positive evaluation of a tourist’s psychological state resulting from his or her travel experience. Satisfaction is an important factor of user behavioral intention [111], and it is an important variable in the success model of information systems. In a previous study [109], AR satisfaction was found to be related to users’ technology behavioral intention. For example, Chung et al. [87] confirmed the positive effect of AR satisfaction on users’ behavioral intention. In a study on the use of AR for cultural heritage tourism in Korea by Chung et al. [87], it was shown that AR satisfaction influenced their behavioral intention. In addition, Jung et al. [88] verified a positive correlation between satisfaction and users’ intention to purchase AR navigation systems. In this study, satisfaction with museum AR guided tour refers to the comprehensive evaluation of tourists on the use of AR technology for the guided tour during travel activities [87]. Based on the above evidence, this study proposed the following hypothesis:

H11: Satisfaction has a positive effect on intention to use AR guided tours.

**Fig 1:**
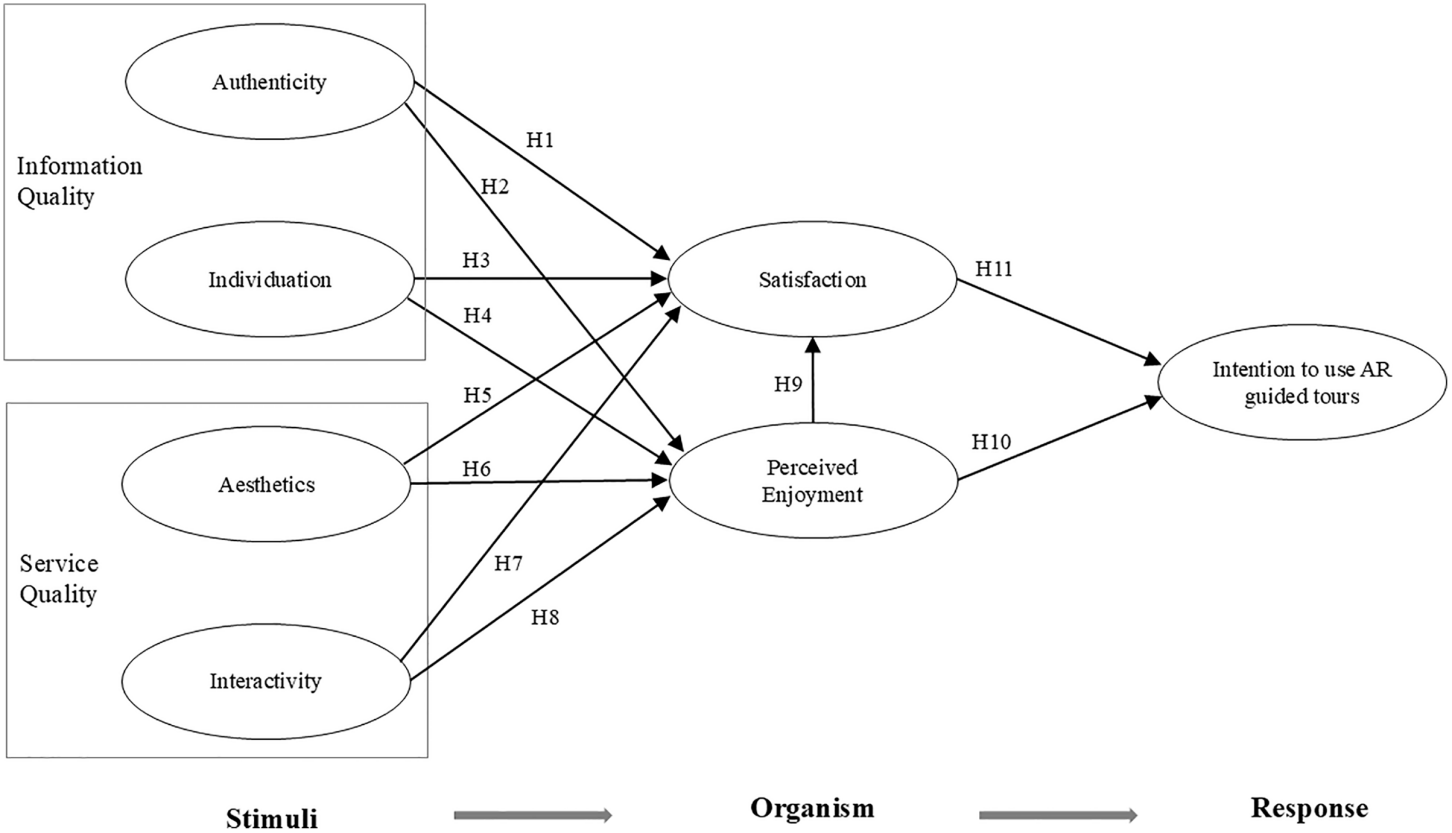
Proposed model.

In summary, this study constructed the theoretical model based on the SOR framework in combination with the theory of ISSM, and proposed the research hypotheses as shown in Fig 1.

## Research methodology

### Research subjects

The research subjects of this study were the users who had experience in visiting museums and were 18 years old and older. The questionnaire was filled out on WJX, one of the largest online questionnaire platforms in China, and WeChat, the largest social networking application in China. The questionnaire can only be completed once per person. The researchers selected suitable respondents from the neighborhood through teachers, classmates and friends, and identified the research subjects with the snow-balling method. The respondents were informed of the purpose and content of the study and volunteered to participate in the study, while those who did not wish to participate in the study were not included in the list of respondents who completed the questionnaire. The ethics application was approved by the School of cultural industries and tourism, Xiamen University of Technology (Date of approval: March 12, 2024). All respondents in this study agreed to participate in this survey and completed the questionnaire voluntarily. The survey was conducted from March 23 to June 21, 2024, and a total of 349 questionnaires were returned, with 324 valid questionnaires.

### Research instrument

The questionnaire was the instrument used in this study. The questionnaire consisted of two parts: the scale (See Appendix A in S1 Appendix) and the personal information. To ensure the validity of the scale, we adapted the scale from the previous literature and combined it with the theme of this study. Specifically, authenticity was adapted from Pietschmann et al. [112], with a total of three questions, such as “the museum AR guided tour provides me with an authentic experience”. Individuation was adapted from Jeong and Shin [110], with a total of four questions, such as “the museum AR guided tour can provide me with individualized tour customization services, the museum AR guided tour can provide dialect navigation, star voice navigation and other special services”. Aesthetics was adapted from Hosany and Witham [113] and Quadri-Felitti et al. [114], with three questions, including “I think the interface design of the museum AR guided tour is coordinated”. Interactivity was adapted from Han et al. [115] and Nguyen, Le and Chau [97], with three questions, such as “I think the museum AR guided tour has good interactive feedback”. Satisfaction was adapted from Kim et al. [116], with three questions, such as “overall, I was satisfied with the museum AR guided tour experience”. Perceived enjoyment was referenced from Rese et al. [117], with three questions, such as “I don’t feel bored when using the museum AR guided tour product”. The intention to use AR guided tour was adapted from Jung et al. [33], with 3 questions, such as “I would recommend the museum AR guided tour to my family and friends”. The scale was based on a 7-point Likert scale for measurement. In addition to age, gender and education level, the personal information section collected the respondents’ current occupation and the number of times they had visited the museum in the previous year. In order to make it easy for the respondents to understand the topic of this study, we gave a general introduction to the museum AR tour at the beginning of the questionnaire. That is, the museum Augmented Reality technology tour refers to the use of augmented reality (AR) technology through mobile devices or dedicated hardware devices. Integrating virtual information with the real exhibits and spatial environment of the museum in real time enables visitors to obtain exhibit information in a more intuitive and vivid way, thereby providing them with an interactive and personalized visiting experience.

### Statistical analysis method

After the questionnaires were collected, the researchers first used SPSS 25.0 to conduct basic analysis of the data such as missing values and descriptive statistics. Then, partial least squares structural equation modeling (PLS-SEM) was used to analyze the model and test the research hypotheses. PLS-SEM was chosen because it can be used to evaluate more complex conceptual models [118] and there are no mandatory requirements on the distribution of questionnaire data [119]. In addition, Hair et al. [120] stated that this method is suitable for theory analysis and test in exploratory studies. The researchers strictly followed the analytical approach of PLS-SEM to analyze the research model, including the evaluation of the measurement model and the structural model.

### Common method bias

In order to reduce the problem of common method bias in this study, the researchers analyzed the questionnaire data in addition to using an anonymous questionnaire. Podsakoff et al. [121] pointed out that Harman’s one-factor analysis is one of the methods to assess common method bias. When the explanation rate of a single factor is below the threshold of 50%, it means that the study does not have the problem of common method bias [121]. The results of the study showed that the explanation rate of the single factor was 46.484%, which did not exceed the criterion, indicating that the common method bias did not exist.

## Results

### Demography

A total of 349 questionnaires were collected in this study. After 25 invalid questionnaires were excluded, such as those with unanimous answers, 324 valid questionnaires were left. The demographics data of the respondents are shown in Table 1, including 125 males (38.6%) and 199 females (61.4%). The largest number of the respondents were aged 25 ~ 34 years, totaling 111, accounting for 34.3%, followed by 90 respondents 18 ~ 24 years old, and 72 respondents 35 ~ 44 years old. The largest number of the respondents had a bachelor’s degree, with 159 respondents, followed by 98 respondents with a junior college degree. Among the current occupations, 157 respondents were full-time employees, accounting for the highest proportion of 48.5%, followed by 101 respondents who were freelancers. In addition, for the number of visits to the museum in the previous year, it was 1–3 times for 137 respondents and 4–6 times for 109 respondents.

**Table 1:** Demography.

Category	Group	Frequency	Percentage (%)
Gender	Male	125	38.6
	Female	199	61.4
Age	18 ~ 24	90	27.8
	25 ~ 34	111	34.3
	35 ~ 44	72	22.2
	45 ~ 54	35	10.8
	≥55	16	4.9
Education	High school and below	25	7.7
	Junior college	98	30.2
	Bachelor degree	159	49.1
	Master degree or above	42	13.0
Current occupations	Full-time employees	157	48.5
	Freelancers	101	31.1
	Retire	10	3.1
	Student	56	17.3
The number of visits to the museum in the previous year	1 ~ 3 times	137	42.3
	4 ~ 6 times	109	33.7
	7 ~ 9 times	61	18.8
	≥10 times	17	5.2

### Measurement model

The reliability of the measurement model was assessed by the researchers through the index reliability, internal consistency, convergent validity and discriminant validity of the variables. Table 2 shows that the maximum Cronbach’s alpha value of this study’s dimensions was 0.796, the minimum value was 0.717, and the Cronbach’s alpha of all variables reached the threshold of 0.7 [120], which indicated that the internal consistency of the variables met the requirements of reliability. Fornell and Larcker [122] pointed out that if the factor loading of each measurement index is more than 0.5, it means that the study has index reliability. Table 1 shows that the minimum value of the factor loading of measurement indexes was at 0.736, which indicated that the index reliability met the requirements. Hair et al. [119] stated that when the square root of the AVE is higher than the value of the correlation coefficient of the dimensions, the scale has discriminant validity. The test of the discriminant validity was conducted in this study and the results in Table 3 showed that the square root of AVE for all the variables exceeded the value of correlation coefficient of the dimensions, which indicated that the discriminant validity was achieved. Nunnally [123] stated that when the average variance extracted (AVE) of a variable exceeds 0.5, it indicates that the construct has convergent validity. Table 2 shows that the lowest value of AVE of a variable is 0.620, which indicates that the variables has convergent validity.

**Table 2:** Construct reliability and validity.

Variable	Items	Loading	Cronbach’s Alpha	CR	AVE
Aesthetics	AE1	0.827	0.765	0.865	0.681
	AE2	0.825			
	AE3	0.823			
Authenticity	AU1	0.809	0.741	0.852	0.658
	AU2	0.843			
	AU3	0.781			
Individuation	IND1	0.780	0.796	0.867	0.620
	IND2	0.783			
	IND3	0.799			
	IND4	0.787			
Intention to Use	IUT1	0.814	0.758	0.861	0.674
	ITU2	0.806			
	ITU3	0.843			
Interactivity	INT1	0.819	0.721	0.843	0.641
	INT2	0.820			
	INT3	0.762			
Perceived Enjoyment	PE1	0.800	0.737	0.851	0.655
	PE2	0.818			
	PE3	0.809			
Satisfaction	SA1	0.736	0.717	0.842	0.640
	SA2	0.829			
	SA3	0.831			

**Table 3:** Fornell-larcker criterion.

	Aesthetics	Authenticity	Individuation	Intention to Use	Interactivity	Perceived Enjoyment	Satisfaction
Aesthetics	**0.825**						
Authenticity	0.711	**0.811**					
Individuation	0.754	0.735	**0.788**				
Intention to Use	0.599	0.572	0.599	**0.821**			
Interactivity	0.732	0.689	0.723	0.596	**0.801**		
Perceived Enjoyment	0.625	0.595	0.613	0.760	0.604	**0.809**	
Satisfaction	0.672	0.622	0.710	0.674	0.670	0.692	**0.800**

Note, The bold number is the square root of AVE.

### Structural model

Hair et al. [119] suggested that Stone-Geisser’s Q 2 can be used to assess the prediction relevance of a model, and when Q 2 is greater than 0, the exogenous variables have prediction relevance. As shown in Table 4, the Q 2 of each dimension was greater than 0, indicating that the model had prediction relevance. Falk and Miller [124] stated that when the R2 value of the endogenous dimension is greater than 10%, it means that the research model has explanatory power. As shown in Table 5, the value of R2 of use intention was 0.620, and the results showed that the R2 values of this study all reached the level, indicating that the research model had explanatory power. Tenenhaus et al. [125] pointed out that when the Goodness of Fit is higher than 0.36, it indicates that the research model has high fit. In this study, the Goodness of Fit value was assessed to be 0.612, which is higher than the criterion for high fit of 0.36. In addition, Tenenhaus et al. [125] stated that the good fit of the research model is also indicated when the Standardized Root Mean Square Residual (SRMR) is less than 0.08. The result showed that the value of SRMR was 0.063, which met the requirement and indicated that the model in this study had good fit.

**Table 4:** Variable Prediction Correlation.

	Q² (=1-SSE/SSO)
Intention to Use	0.410
Perceived Enjoyment	0.300
Satisfaction	0.393

**Table 5:** R Square values.

	R Square
Intention to Use	0.620
Perceived Enjoyment	0.469
Satisfaction	0.633

**Fig 2:**
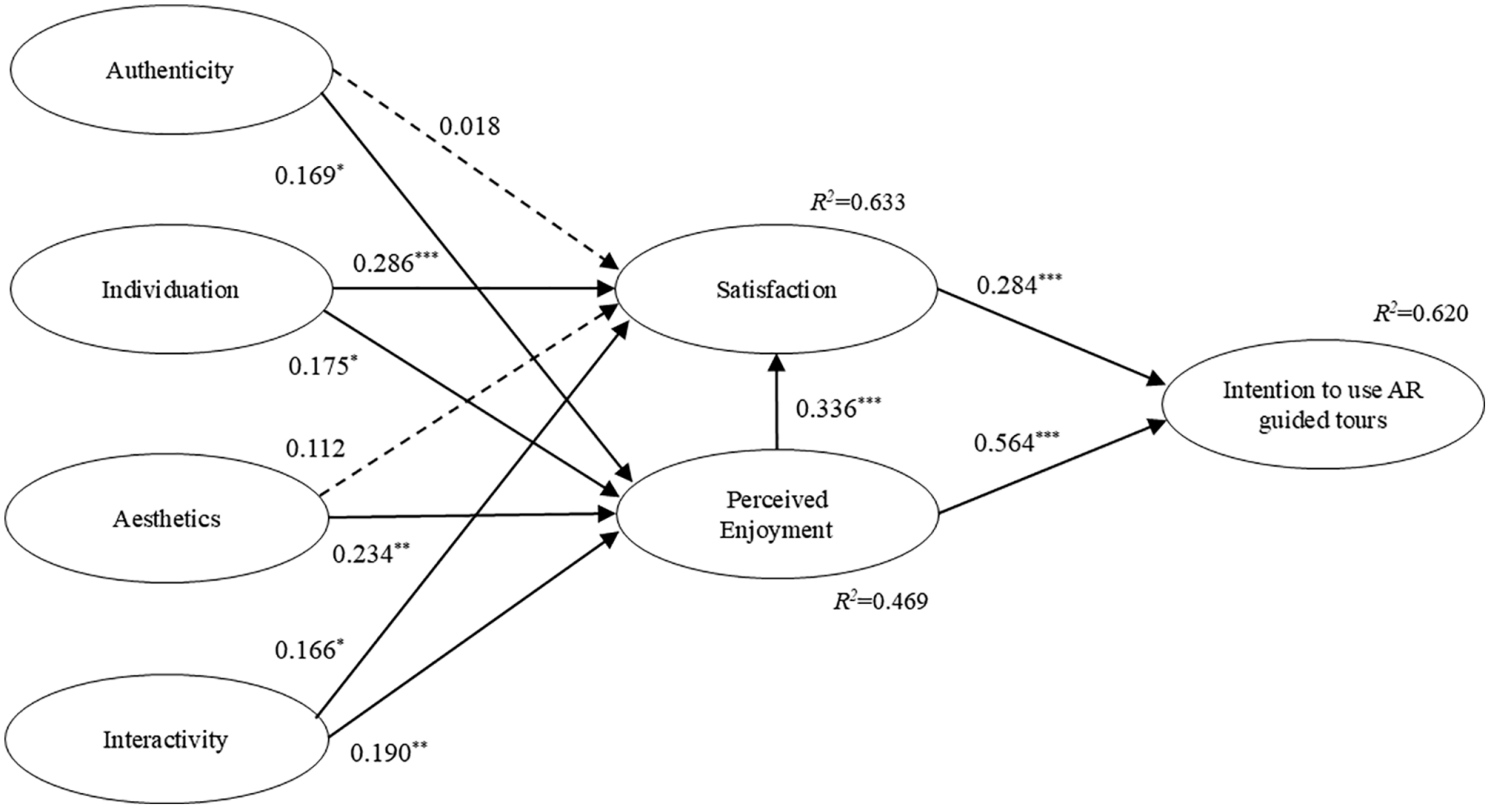
Results of model analysis.

In this study, the path coefficients of the research model were evaluated using the bootstrap method with 5000 repetitive samples [126], and the path coefficients and their significance values are shown in Fig 2 and Table 6. The results showed that authenticity positively affected perceived enjoyment (β = 0.169, t = 2.370, p < 0.05), so H2 was supported. However, authenticity was not significant for satisfaction (β = 0.018, t = 0.259), and H1 was not supported. Individuation positively affected satisfaction (β = 0.286, t = 4.054, p < 0.001) and perceived enjoyment (β = 0.175, t = 2.474, p < 0.05), so H3 and H4 were supported. Aesthetics positively affected perceived enjoyment (β = 0.234, t = 3.277, p < 0.01), so H6 was supported, but the effect of aesthetics on satisfaction was not significant (β = 0.112, t = 1.857), so H5 was not supported. Interactivity was confirmed to have a direct positive effect on satisfaction (β = 0.166, t = 2.579, p < 0.05) and perceived enjoyment (β = 0.190, t = 2.820, p < 0.01), thus H7 and H8 were supported. The results also showed that perceived enjoyment had a direct positive effect on satisfaction (β = 0.336, t = 6.842, p < 0.001) and intention to use the AR guided tour (β = 0.564, t = 11.041, p < 0.001), so H9 and H10 were supported. In addition, our results also showed that satisfaction had a direct positive effect on intention to use AR guided tour (β = 0.284, t = 5.196, p < 0.001), thus, H11 was supported.

**Table 6:** Hypothesis validation results.

Path	Original Sample	T Statistics	P Values	Result
H1: Authenticity - > Satisfaction	0.018	0.259	0.796	Unsupported
H2: Authenticity - > Perceived Enjoyment	0.169	2.370	0.018	Supported
H3: Individuation - > Satisfaction	0.286	4.054	0.000	Supported
H4: Individuation - > Perceived Enjoyment	0.175	2.474	0.013	Supported
H5: Aesthetics - > Satisfaction	0.112	1.857	0.063	Unsupported
H6: Aesthetics - > Perceived Enjoyment	0.234	3.277	0.001	Supported
H7: Interactivity - > Satisfaction	0.166	2.579	0.010	Supported
H8: Interactivity - > Perceived Enjoyment	0.190	2.820	0.005	Supported
H9: Perceived Enjoyment - > Satisfaction	0.336	6.842	0.000	Supported
H10: Perceived Enjoyment - > Intention to Use	0.564	11.041	0.000	Supported
H11: Satisfaction - > Intention to Use	0.284	5.196	0.000	Supported

## Discussion and contribution

### Discussion

AR and other technological innovations and applications have brought new changes to museum tourism services [127]. In the past, the static exhibition guided tour of museums made tourists only watch and browse statically through the windows, and this process is simple and lack of interactive entertainment [128], which makes it not easy for the knowledge related to the collections to be understood and absorbed by tourists. The current service model of museums has shifted from “object-centered” to “visitor-experience-centered” [129]. Therefore, understanding how AR affects user satisfaction and behavioral intention is extremely important for AR developers and AR application units to develop marketing strategies to enrich user experience [130]. Under the framework of SOR, this study explored the effects with authenticity, individuation, aesthetics and interactivity as the stimuli, perceived enjoyment and satisfaction as the organism, and the intention to use AR guided tours as the response based on the ISSM. The study revealed the factors affecting the intention to use AR guided tours, and the results can provide valuable references for museum administrators and AR guided tour developers.

Our results showed that authenticity positively influenced perceived enjoyment, a result also confirmed in Lu et al. [76] on camping tourism. As Jiménez-Barreto et al. [131] pointed out, the experience of visiting a tourist destination combines various experiential value elements in a virtual environment, which conveys rich authenticity content of the object to the visitors and brings pleasure to the users. Therefore, when museums apply AR for tour guiding, they should present the original authenticity of the collection information as much as possible, and present the rich information of the collection. However, the positive effect of authenticity on satisfaction in this study was not significant, which is inconsistent with the findings of Yi et al. [132], which confirmed that authenticity was one of the driving factors affecting tourism satisfaction. This may be due to the fact that Yi et al. [132] focused on the object of cultural heritage sites, whereas in our study, although in a museum context, the users may have been more concerned with the AR guided tours themselves, and they were already aware of the imperfect authenticity of the information in the current AR-constructed virtual contexts. At the same time, as purposeful and 18 years old or older museum visitors, they were more or less aware of the museum they were going to visit and its contents, and had some discernment ability. They may not have such high expectations for the authenticity presented by using AR for navigation. Furthermore, the AR guided tour service that this study focuses on in the real spatial context of museums has already placed visitors in a certain real situation, and they may already have a certain sense of immersion in their hearts. To sum up, they did not care about the authenticity of the AR guided tour program, and naturally, authenticity did not have an impact on their satisfaction.

Our study showed that individuation positively affected users’ perceived enjoyment. This finding is consistent with the results of Zhang et al. [78], this was confirmed in the research of mobile platforms from the perspective of application satisfaction and usage as in Huang and Zhou [133]. Meanwhile, there was a positive correlation between individuation and satisfaction, which confirmed the findings of [79]. In other words, the individualized content presentation of museum AR guide tour system will bring positive enjoyment and satisfaction to users. Individuation represents a matching tool between the platform content information and users’ needs [134]. In museums, there are a lot of collections, but visitors have limited time, which makes it difficult for users to obtain the content and information they need, and individualized tour information effectively solves the problem. Therefore, an excellent museum AR guided tour system should be user-oriented, use multi-technology integration based on AR technology to provide individualized content information for visitors, improve the accuracy of the recommended guide information, and provide individualized and featured content to meet the content needs of different visitors. The personalization of AR guided Tours in museums should start from the perspective of tourists and take their needs as the core. For example, individualized information should be provided to meet the needs of student visitors or elderly visitors. In addition, in the individualized AR guided tour settings, museums can also try to provide individualized and targeted tour routes according to the age and background of the tourist users and their previous tour records, so as to greatly reduce the unnecessary time of the users in searching for information and make the tourist users feel happy and satisfied during the tour process.

Our results confirmed that aesthetics was an important predictor of perceived enjoyment. This finding is consistent with the results of a previous study [87]. It suggests that the aesthetics of museum AR guided tours can generate positive emotions for AR guided tours [135]. Therefore, with the widespread use of AR in museums and cultural tourist attractions, aesthetic experience should be taken into consideration to provide tourists with a satisfying tourist visit experience [88]. Because museum exhibitions are emotional, they can trigger users’ aesthetic experiences [91]. Considering that aesthetics is to gain comfort by appealing to the visual appeal of the senses [88], the aesthetics of AR in museums can be seen as whether or not it provides tourists with an aesthetically pleasing aesthetic experience that immerses the user in the aesthetic environment presented by the AR [136]. Therefore, in order for visitors to form a positive view of the museum and feel it attractive, the interface and other carriers should be presented in a visually appealing way in the design and development of the AR interface to meet the aesthetic requirements of the users, so as to enhance the perceived enjoyment of the visitors to the AR guided tour. For example, the data information of the collection is presented in a layered and iterative way, attention is paid to the layout of the information classification, the design of fonts and colors is coordinated as much as possible and matched with the age and style of the collection, and the important information is highlighted in the design, and so on. However, aesthetics is not significant for satisfaction, and the reasons may be multi-faceted. For example, this study focuses more on the aesthetics of the AR technology system itself, including the interface, layout, etc., rather than the aesthetics of the exhibition and design of the museum. Therefore, the focus of the users in this study is also on the aesthetic aspect of the AR technology system, etc. In the immersive learning fields of museums and art galleries, they may be more focused on the museum content (such as collections, etc.) displayed by the AR technology system. As a result, they may pay less attention to the interface aesthetics of the AR technology system, etc. Therefore, the research results are not significant.

As an informal educational place, museums also have certain educational and popularization functions. When AR is applied to museum education and tourism, it is suitable for advancing visitors’ experience of perceived enjoyment [137]. Just as the research of Wangming Hu and Hyunsuk Han [94] confirmed, the use of augmented reality technology in museums can enhance more interactivity and entertainment for young users. Our study showed that when museum AR guided tours brought interactivity to users, it led to a different kind of enjoyment perception to users. This finding is similar to that of Nguyen et al. [97]. As one of the important medium features of AR, interactivity is a feature that allows visitors to manipulate the virtual environment to a certain extent [138], and it is one of the key determinants of enjoyment in interactive systems [139], which was verified in our study. Therefore, in order to ensure a positive user experience and increase the satisfaction with museum AR guided tours, immersive and interactive experiences of AR guided tours should be enriched by setting up interactive modes of AR and user participation during the process of viewing or listening to museum collections, such as setting up question and answer interactions with tourists in the process of AR guided tours. This will enrich the user’s sense of personal participation, enhance visitors’ perceived enjoyment of the AR guided tour, and improve user satisfaction.

As an entertainment medium, AR can provide users with entertainment experiences, including exciting consumer activity experiences [137]. Nechita and Rezeanu [140], from the perspective of entertainment, confirmed that the sensory stimulation shaped by AR would enhance the relationship between young users and museums. This study showed that perceived enjoyment positively influenced user satisfaction and behavioral intention, as also verified by Verhagen et al. [104] and Leveau and Camus [107], respectively. Furthermore, the research on the art gallery environment by Hyowon and Jungkun [92] also shows that hedonism has a significant impact on user satisfaction. Therefore, this study and the previous related studies mentioned above, this suggests that users can obtain enjoyment through aesthetic, interactive and other sensory stimuli, which in turn increases the value of experiential satisfaction [113]. In addition, our results showed that satisfaction positively influenced users’ intention to use AR guided tours, which is consistent with the results of Peng et al.’s [141] study on customers’ intention to make purchases in stores. This discovery was also confirmed in the research of Li, Tu [1] on the reference museum using wearable AR technology. This suggests that AR, which enhances the richness of the tour experience for tourists, will cause them to show certain emotional responses, such as satisfaction [142], and this will increase their intention to use the AR guided tour.

### Contribution

The theoretical contributions of this study are as follows. First, the study con-structed and empirically demonstrated a technology acceptance model of museum AR guided tours, which enriched the theory of user behavior and provided theoretical support for the user behavior of museum AR guided tours. Then, based on the theoretical foundation of the SOR framework and the ISSM, the study expanded the SOR theory and the ISSM, and enriched the application areas of these theories. Third, the study confirmed the significant impact of individuation and authenticity on perceived enjoyment, which in turn affected the intention to use AR guided tours. This revealed that users seek authenticity and individuation in the quality of AR content in museums, as well as the positive relationship between users’ intrinsic positive emotions and their behavioral responses, which makes up for the lack of consideration of intrinsic emotional changes in the users’ internal moods and the relationship between moods and users’ behaviors. Finally, with the advancement of information technology and the changing needs of user experience, digital museums are being constructed. Although there are studies on intelligent technologies in museums, however, the research on usage intention carried out in the context of AR guided Tours in museums is still slightly insufficient. This study is an enrichment of the exploration in this field, and it provides valuable references to the study of the behavior of users of AR guided tours in museums.

The practical contributions of this study are mainly in the following four aspects. First, the findings of this study can provide an important reference for system development to the developers and providers of AR guided tour systems. This means that information can be provided according to different needs of visitors, which means that technicians should organize the tour information according to the user’s background and needs. Meanwhile, developers and designers should make individualized recommendations based on visitors’ information preferences, thus providing visitors with satisfaction and enjoyment. Second, museum administrators and marketers should grasp the positive emotional needs of visitors and create multi-channel communication and publicity. On the basis of the findings of this study, combined with the concept of visitor-centered service, they may strengthen the promotion of some features and functions of their AR guided tours, including individuation, interactivity and aesthetics, in order to attract tourists to visit the museums. Third, based on the results of the study, the stimulus factors that influence the psychological and behavioral intentions of visitors should be strengthened. Attention should be paid to designing tour interfaces that meet the aesthetics of different users, offering multi-sensory tour inter-actions, and providing authentic and rich information about the collections, so as to create a pleasurable tour experience and satisfy the diversified needs of tourists. Fourth, it is necessary to ensure that the design of the AR navigation system has a high level of information quality and service quality, so as to bring tourists a sense of pleasure and satisfaction, thereby influencing users’ decisions and attracting tourists. The research results show that when museum AR guided Tours have rich interactive methods and provide higher service quality, users’ experience will be better and their intention to use will be stronger. Therefore, attempts can be made to enhance the interactivity of users through forms such as text, voice, pictures, videos and virtual interaction, improve the guiding experience of users, promote the integration of museum knowledge and culture, and thereby better display the connotation of museum exhibits and disseminate their cultural information.

## Conclusion and future directions

### Conclusion

As a digital technology, AR provides a new way of museum guided tours for visitors. This study focused on museum AR guided tours and explored users’ behaviors. In this study, authenticity and individuation were taken as information quality, aesthetics and interactivity were taken as service quality, and they were all considered as the stimuli, while perceived enjoyment and satisfaction were taken as the organisms for the exploration of their effects on the response of intention to use AR guided tours. The results showed that perceived enjoyment was directly influenced by authenticity, individuation, aesthetics and interactivity, while individuation, interactivity and perceived enjoyment were the antecedent influencing factor of satisfaction, and perceived enjoyment and satisfaction directly affected the intention to use AR guided tours. This study has enriched the theory of museum user behavior, provided valuable insights for museum administrators, tourism publicity promoters and AR guided tour developers and designers, and offered new perspectives for the construction of “visitor-centered” digital museums to promote the healthy and sustainable development of museum tourism.

## Future directions

There are some shortcomings in this study, which can provide directions for sub-sequent research. First, the research subjects of this study were Chinese tourists, so the results of this study may not be applicable to other countries or regions. Visitors from different countries or regions may have different perceptions of the technology of museum AR guided tours due to cultural differences and different requirements. Therefore, follow-up research should include visitors from more countries or regions for comparative studies. Then, the information of this study was obtained from a cross-sectional survey, which may affect the results to some extent. A longitudinal study should be conducted in the future to obtain richer information and results. And, users’ new technology use behavior is complex, and it is affected by many factors. This study has explored some factors, but it may still be incomplete. For example, due to the differences in age, income and location of tourists, these factors may have a moderating effect on their technology use behavior to some extent. Therefore, follow-up studies can try to incorporate more dimensional factors, or analyze and explore a specific factor in a multi-dimensional way, in order to gain a more systematic insight into the decision-making process of users’ technology use and its influencing factors.

## Supporting information

S1 Appendix Appendix A Questionnaire scale. (DOC)

## References

[1] Li J, Tu X-F, Nie J-W, Ye J, Lv C. Visitors’ Acceptance of Wearable AR Technology in Museums. Sage Open. 2024;14(4). doi: 10.1177/21582440241303507

[2] Pai CK, Liu YM, Kang S, Dai AN. The Role of Perceived Smart Tourism Technology Experience for Tourist Satisfaction, Happiness and Revisit Intention. Sustainability. 2020;12(16).

[3] Nizar NNM, Rahmat MK. Examining the museum visitors use of mobile technology through technology acceptance model (TAM). Journal of Tourism, Hospitality, and Event Management. 2018;3(11):14–24.

[4] Orthwein M. 360 “Beyond the Screen” – Immersive, Stereo-Surround Projects in cooperation with Museum of Ancient Seafaring in Mainz. Electronic Visualisation and the Arts (EVA 2015); 2015.

[5] Annechini C, Menardo E, Hall R, Pasini M. Aesthetic attributes of museum environmental experience: A pilot study with children as visitors. Frontiers in Psychology. 2020. 10.3389/fpsyg.2020.508300 PMC7604523 33192758

[6] Tussyadiah IP, Jung TH, tom Dieck MC. Embodiment of Wearable Augmented Reality Technology in Tourism Experiences. Journal of Travel Research. 2017;57(5):597–611. doi: 10.1177/0047287517709090

[7] Keil J, Edler D, Dickmann F. Preparing the HoloLens for user Studies: an Augmented Reality Interface for the Spatial Adjustment of Holographic Objects in 3D Indoor Environments. KN J Cartogr Geogr Inf. 2019;69(3):205–15. doi: 10.1007/s42489-019-00025-z

[8] Zhou Y, Chen J, Wang M. A meta-analytic review on incorporating virtual and augmented reality in museum learning. Educational Research Review. 2022;36:100454. doi: 10.1016/j.edurev.2022.100454

[9] Grammatikopoulou A, Grammalidis N. Artful—An AR social self-guided tour app for cultural learning in museum settings. Information. 2023;14(3):33.

[10] Nisi V, Dionisio M, Barreto M, Nunes N. A Mixed Reality neighborhood tour: Understanding visitor experience and perceptions. Entertainment Computing. 2018;27:89–100. doi: 10.1016/j.entcom.2018.04.002

[11] Han DID, Dieck TMC, Jung T. Augmented Reality Smart Glasses (ARSG) Visitor Adoption in Cultural Tourism. Leisure Studies. 2019;:1–16.

[12] Rahaman H, Champion E, Bekele M. From photo to 3D to mixed reality: A complete workflow for cultural heritage visualisation and experience. Digital Applications in Archaeology and Cultural Heritage. 2019;13.

[13] Garau C. From Territory to Smartphone: Smart Fruition of Cultural Heritage for Dynamic Tourism Development. Planning Practice & Research. 2014;29(3):238–55. doi: 10.1080/02697459.2014.929837

[14] Moorhouse N, Dieck MCT, Jung T. An experiential view to children learning in museums with augmented reality. Museum Management and Curatorship. 2019;34(4):402–18.

[15] tom Dieck MC, Jung TH, tom Dieck D. Enhancing art gallery visitors’ learning experience using wearable augmented reality: generic learning outcomes perspective. Current Issues in Tourism. 2016;21(17):2014–34. doi: 10.1080/13683500.2016.1224818

[16] Carrozzino M, Bergamasco M. Beyond virtual museums: Experiencing immersive virtual reality in real museums. Journal of Cultural Heritage. 2010;11(4):452–8. doi: 10.1016/j.culher.2010.04.001

[17] Jung TH, Lee H, Chung N, tom Dieck MC. Cross-cultural differences in adopting mobile augmented reality at cultural heritage tourism sites. IJCHM. 2018;30(3):1621–45. doi: 10.1108/ijchm-02-2017-0084

[18] Arrigoni G, Schofield T, Pisanty DT. Framing collaborative processes of digital transformation in cultural organisations: from literary archives to augmented reality.

[19] Dieck T, Jung TH. Value of augmented reality at cultural heritage sites: A stakeholder approach. Journal of Destination Marketing & Management. 2017. doi: S2212571X16300774

[20] Bernik A, Vusi D, Kober D. Implementation of Augmented Reality Application and Computer Graphics: The Case of the Stolen Paintings. Technical Gazette/ Tehnički Vjesnik. 2019;26(6).

[21] Van Nguyen S, Le ST, Tran MK, Tran HM. Reconstruction of 3D digital heritage objects for VR and AR applications. Journal of Information and Telecommunication. 2021;6(3):254–69. doi: 10.1080/24751839.2021.2008133

[22] Gimeno J, Portalés C, Coma I, Fernández M, Martínez B. Combining traditional and indirect augmented reality for indoor crowded environments. A case study on the Casa Batlló museum. Computers & Graphics. 2017;69:92–103. doi: 10.1016/j.cag.2017.09.001

[23] Sheng C-W, Chen M-C. A study of experience expectations of museum visitors. Tourism Management. 2012;33(1):53–60. doi: 10.1016/j.tourman.2011.01.023

[24] Kim J, Ahn K, Chung N. Examining the factors affecting perceived enjoyment and usage intention of ubiquitous tour information services: A service quality perspective. Asia Pac J Tour Res. 2013;18(6):1–20.

[25] Mason M. The Dimensions of the Mobile Visitor Experience: Thinking beyond the Technology Design. The International Journal of the Inclusive Museum. 2013;5(3):51–72. doi: 10.18848/1835-2014/cgp/v05i03/44404

[26] Han S, Yoon JH, Kwon J. Impact of experiential value of augmented reality: The context of heritage tourism. Sustainability. 2021;13(8).

[27] Trunfio M, Lucia MD, Campana S, Magnelli A. Innovating the cultural heritage museum service model through virtual reality and augmented reality: the effects on the overall visitor experience and satisfaction. Journal of Heritage Tourism. 2021;17(1):1–19. doi: 10.1080/1743873x.2020.1850742

[28] Bae S, Jung TH, Moorhouse N, Suh M, Kwon O. The influence of mixed reality on satisfaction and brand loyalty in cultural heritage attractions: A brand equity perspective. Sustainability. 2020;12(7).

[29] King E, Smith MP, Wilson PF, Williams MA. Digital responses of UK museum exhibitions to the COVID‐19 crisis, March – June 2020. Curator: The Museum Journal. 2020. 10.1111/cura.12413 PMC8251409 34230675

[30] Giannini T, Bowen JP. Museums and digitalism. Museums and digital culture. Springer. 2019. p. 27–46.

[31] Olya H, Jung TH, Tom Dieck MC, Ryu K. Engaging visitors of science festivals using augmented reality: asymmetrical modelling. IJCHM. 2020;32(2):769–96. doi: 10.1108/ijchm-10-2018-0820

[32] Pallud J, Straub DW. Effective website design for experience-influenced environments: The case of high culture museums. Information & Management. 2014;51(3):359–73. doi: 10.1016/j.im.2014.02.010

[33] Jung T, Dieck MCT, Lee H, Chung N. Relationships among beliefs, attitudes, time resources, subjective norms, and intentions to use wearable augmented reality in art galleries. Sustainability. 2020;12(20).

[34] Aristidou M, Stylianou-Lambert T. VR/AR artworks in the museum: redefining preservation through collaboration. Convergence. 2024;30(5):1575–95.

[35] Van Winkle CM, Bueddefeld JNH, Halpenny EA, MacKay KJ. The unified theory of acceptance and use of technology 2: understanding mobile device use at festivals. Leisure Studies. 2019;38(5):634–50. doi: 10.1080/02614367.2019.1618895

[36] The DeLone and McLean Model of Information Systems Success: A Ten-Year Update. Journal of Management Information Systems. 2003;19(4):9–30. doi: 10.1080/07421222.2003.11045748

[37] Loureiro SMC, Guerreiro J, Ali F. 20 years of research on virtual reality and augmented reality in tourism context: A text-mining approach. Tourism Manage. 2020;77.

[38] Mehrabian A, Russell JA. An approach to environmental psychology. Cambridge, MA: Massachusetts Institute of Technology. 1974.

[39] Song ZJ, Liu C, Shi R. How do fresh live broadcast impact consumers’ purchase intention? Based on the SOR theory. Sustainability. 2022;14(21).

[40] Zhuang X, Hou X, Feng Z, Lin Z, Li J (Justin). Subjective norms, attitudes, and intentions of AR technology use in tourism experience: the moderating effect of millennials. Leisure Studies. 2020;40(3):392–406. doi: 10.1080/02614367.2020.1843692

[41] Scarles C, Treharne H, Casey M, Abidin HZ. Micro-mobilities in curated spaces: agency, autonomy and dwelling in visitor experiences of augmented reality in arts and heritage. Mobilities. 2020;15(6):776–91.

[42] Chen H, Ryan C. Transforming the museum and meeting visitor requirements: The case of the Shaanxi History Museum. Journal of Destination Marketing & Management. 2020;18:100483. doi: 10.1016/j.jdmm.2020.100483

[43] Wu Y, Jiang Q, Liang H, Ni S. What Drives Users to Adopt a Digital Museum? A Case of Virtual Exhibition Hall of National Costume Museum. Sage Open. 2022;12(1). doi: 10.1177/21582440221082105

[44] Wang Z. Self-cognition in the construction of digital museums: A study based on the collection of famous paintings in the palace museum. Open J Soc Sci. 2018;6(11):293–300.

[45] Saiki D, Robbins A. Trends in information categories on costume and textile collection web sites. The Electronic Library. 2008;26(6):821–32. doi: 10.1108/02640470810921600

[46] Martin K, Ko H-S. Virtual Historic Costume across Cultures and Disciplines. In: 2009 15th International Conference on Virtual Systems and Multimedia, 2009. 104–8. http://dx.doi.org/10.1109/vsmm.2009.21doi: 10.1109/vsmm.2009.21

[47] Perez-Vega R, Kaartemo V, Lages CR, Borghei Razavi N, Männistö J. Reshaping the contexts of online customer engagement behavior via artificial intelligence: A conceptual framework. Journal of Business Research. 2021;129:902–10. doi: 10.1016/j.jbusres.2020.11.002

[48] Gabriel A, Ajriya AD, Fahmi CZN, Handayani PW. The influence of augmented reality on E-commerce: A case study on fashion and beauty products. Cogent Business & Management. 2023;10(2). doi: 10.1080/23311975.2023.2208716

[49] Jeong Y, Kim E, Kim SK. Understanding active sport tourist behaviors in small-scale sports events: stimulus-organism-response approach. Sustainability. 2020;12(19).

[50] Bigne E, Chatzipanagiotou K, Ruiz C. Pictorial content, sequence of conflicting online reviews and consumer decision-making: The stimulus-organism-response model revisited. Journal of Business Research. 2020;115:403–16. doi: 10.1016/j.jbusres.2019.11.031

[51] Shim M, Jo HS. What quality factors matter in enhancing the perceived benefits of online health information sites? Application of the updated DeLone and McLean Information Systems Success Model. Int J Med Inform. 2020;137:104093. doi: 10.1016/j.ijmedinf.2020.104093 32078918

[52] Al-Fraihat D, Joy M, Masa’Deh RE, Sinclair J. Evaluating e-learning systems success: An empirical study. Comput Hum Behav. 2020;102(Jan.):67–86.

[53] Lowry PB, Karuga GG, Richardson VJ. Assessing leading institutions, faculty, and articles in premier information systems research journals. Communications of the Association for Information Systems. 2007;20(16):142–203.

[54] Duong CD. Applying the stimulus-organism-response theory to investigate determinants of students’ social entrepreneurship: moderation role of perceived university support. SEJ. 2023;19(2):167–92. doi: 10.1108/sej-10-2022-0091

[55] Al-Sulaiti I. Mega shopping malls technology-enabled facilities, destination image, tourists’ behavior and revisit intentions: Implications of the SOR theory. Front Environ Sci. 2022;10. doi: 10.3389/fenvs.2022.965642

[56] Guo J, Li Y, Xu Y, Zeng K. How live streaming features impact consumers’ purchase intention in the context of cross-border e-commerce? A research based on SOR theory. Frontiers in Psychology. 2021. 10.3389/fpsyg.2021.767876 PMC8600228 34803849

[57] Baker J, Parasuraman A, Grewal D, Voss GB. The Influence of Multiple Store Environment Cues on Perceived Merchandise Value and Patronage Intentions. Journal of Marketing. 2002;66(2):120–41. doi: 10.1509/jmkg.66.2.120.18470

[58] Kim MJ, Lee C-K, Jung T. Exploring Consumer Behavior in Virtual Reality Tourism Using an Extended Stimulus-Organism-Response Model. Journal of Travel Research. 2018;59(1):69–89. doi: 10.1177/0047287518818915

[59] Caboni F, Hagberg J. Augmented reality in retailing: a review of features, applications and value. IJRDM. 2019;47(11):1125–40. doi: 10.1108/ijrdm-12-2018-0263

[60] de Amorim IP, Guerreiro J, Eloy S, Loureiro SMC. How augmented reality media richness influences consumer behaviour. Int J Consumer Studies. 2022;46(6):2351–66. doi: 10.1111/ijcs.12790

[61] Choi W, Rho MJ, Park J, Kim K-J, Kwon YD, Choi IY. Information system success model for customer relationship management system in health promotion centers. Healthc Inform Res. 2013;19(2):110–20. doi: 10.4258/hir.2013.19.2.110 23882416 PMC3717434

[62] Zhao Y, Deng SL, Zhou RX. Understanding mobile library apps continuance usage in China: A theoretical framework and empirical study. Libri. 2015;65(3):161–73.

[63] Liu I-F, Chen MC, Sun YS, Wible D, Kuo C-H. Extending the TAM model to explore the factors that affect Intention to Use an Online Learning Community. Computers & Education. 2010;54(2):600–10. doi: 10.1016/j.compedu.2009.09.009

[64] Zafar AU, Qiu J, Li Y, Wang J, Shahzad M. The impact of social media celebrities’ posts and contextual interactions on impulse buying in social commerce. Computers in Human Behavior. 2019;:106178.

[65] Rhee CE, Choi J. Effects of personalization and social role in voice shopping: An experimental study on product recommendation by a conversational voice agent. Comput Hum Behav. 2020;:106359.

[66] Penrose J. Authenticity, authentication and experiential authenticity: telling stories in museums. Social & Cultural Geography. 2018;21(9):1245–67. doi: 10.1080/14649365.2018.1550581

[67] Wu H-C, Cheng C-C, Ai C-H, Wu T-P. Fast-disappearing destinations: the relationships among experiential authenticity, last-chance attachment and experiential relationship quality. Journal of Sustainable Tourism. 2020;28(7):956–77. doi: 10.1080/09669582.2020.1713799

[68] Dai T, Zheng X, Yan J. Contradictory or aligned? The nexus between authenticity in heritage conservation and heritage tourism, and its impact on satisfaction. Habitat International. 2021;107:102307. doi: 10.1016/j.habitatint.2020.102307

[69] Wang N. Rethinking authenticity in tourism experience. Ann Touris Res. 1999;26(2):349–70.

[70] Zhang T, Yin P, Peng Y. Effect of commercialization on tourists’ perceived authenticity and satisfaction in the cultural heritage tourism context: Case study of Langzhong ancient city. Sustainability. 2021;13.

[71] Zerva K. ‘Chance Tourism’: Lucky enough to have seen what you will never see. Tourist Studies. 2017;18(2):232–54. doi: 10.1177/1468797617723471

[72] Lee C-K, Ahmad MS, Petrick JF, Park Y-N, Park E, Kang C-W. The roles of cultural worldview and authenticity in tourists’ decision-making process in a heritage tourism destination using a model of goal-directed behavior. Journal of Destination Marketing & Management. 2020;18:100500. doi: 10.1016/j.jdmm.2020.100500

[73] Hernández-Mogollon JM, Campón-Cerro AM, Alves H. Authenticity in environmental high-quality destinations: A relevant factor for green tourism demand. Environmental Engineering and Management Journal. 2013;12(10):1961–70.

[74] Hede AM, Garma R, Josiassen A, Thyne M. Perceived authenticity of the visitor experience in museums: Conceptualization and initial empirical findings. European Journal of Marketing. 2014;48(7–8):1395–412.

[75] Engeset MG, Elvekrok I. Authentic concepts: effects on tourist satisfaction. Journal of Travel Research. 2015;54(4):456–66.

[76] Lu J, Wang X, Dai Z, Chen G, Feng Y. Antecedents of customer WOM in glamping: The critical role of original ecology. International Journal of Hospitality Management. 2021;95:102919. doi: 10.1016/j.ijhm.2021.102919

[77] Tussyadiah IP, Wang D, Jung TH, tom Dieck MC. Virtual reality, presence, and attitude change: Empirical evidence from tourism. Tourism Management. 2018;66:140–54. doi: 10.1016/j.tourman.2017.12.003

[78] Zhang W, Leng XM, Liu SY. Research on mobile impulse purchase intention in the perspective of system users during COVID-19. Pers Ubiquitous Comput. 2020. 10.1007/s00779-020-01460-w PMC7486589 32952494

[79] No E, Kim JK. Comparing the attributes of online tourism information sources. Computers in Human Behavior. 2015;50:564–75. doi: 10.1016/j.chb.2015.02.063

[80] Madu CN, M AA. Dimensions of e-quality. International Journal of Quality & Reliability Management. 2022;19(3):246–58.

[81] Häubl G, Trifts V. Consumer Decision Making in Online Shopping Environments: The Effects of Interactive Decision Aids. Marketing Science. 2000;19(1):4–21. doi: 10.1287/mksc.19.1.4.15178

[82] Tam KY, Ho SY. Web personalization as a persuasion strategy: An elaboration likelihood model perspective. Inf Syst Res. 2005;16(3):271–91.

[83] Qian X, Feng H, Zhao G, Mei T. Personalized Recommendation Combining User Interest and Social Circle. IEEE Trans Knowl Data Eng. 2014;26(7):1763–77. doi: 10.1109/tkde.2013.168

[84] Liu Y, Li Q, Edu T, Jozsa L, Negricea IC. Mobile shopping platform characteristics as consumer behavior determinants. APJML. 2019;32(7):1565–87. doi: 10.1108/apjml-05-2019-0308

[85] Pathak B, Garfinkel R, Gopal RD, Venkatesan R, Yin F. Empirical Analysis of the Impact of Recommender Systems on Sales. Journal of Management Information Systems. 2010;27(2):159–88. doi: 10.2753/mis0742-1222270205

[86] Tractinsky N. Toward the study of aesthetics in information technology. In: Proceedings of the International Conference on Information Systems, ICIS 2004, Washington, DC, USA, 2004.

[87] Chung N, Lee H, Kim J-Y, Koo C. The Role of Augmented Reality for Experience-Influenced Environments: The Case of Cultural Heritage Tourism in Korea. Journal of Travel Research. 2017;57(5):627–43. doi: 10.1177/0047287517708255

[88] Jung TH, Bae S, Moorhouse N, Kwon O. The impact of user perceptions of AR on purchase intention of location-based AR navigation systems. Journal of Retailing and Consumer Services. 2021;61:102575. doi: 10.1016/j.jretconser.2021.102575

[89] Tang YM, Lau Y, Ho UL. Empowering Digital Marketing with Interactive Virtual Reality (IVR) in Interior Design: Effects on Customer Satisfaction and Behaviour Intention. JTAER. 2023;18(2):889–907. doi: 10.3390/jtaer18020046

[90] Cyr D, Head M, Ivanov A. Design aesthetics leading to m-loyalty in mobile commerce. Inf Manage. 2006;43(8):950–63.

[91] Bedford L. The art of museum exhibitions: How story and imagination create aesthetic experiences. Routledge. 2016.

[92] Hyowon H, Jungkun P, Tianbao R, Hyunjin K. The role of ambiances and aesthetics on millennials’ museum visiting behavior. Arts & the Market. 2018.

[93] Yang F, Shen F. Effects of Web Interactivity: A Meta-Analysis. Communication Research. 2017;45(5):635–58. doi: 10.1177/0093650217700748

[94] Hu W, Han H, Wang G, Peng T, Yang Z. Interactive Design and Implementation of a Digital Museum under the Background of AR and Blockchain Technology. Applied Sciences. 2023;13(8):4714. doi: 10.3390/app13084714

[95] Nah FFH, Eschenbrenner B, Wester D. Enhancing brand equity through flow and telepresence: A comparison of 2D and 3D virtual worlds. Management Information Systems Quarterly. 2011;35.

[96] Kim D, Ko YJ. The impact of virtual reality (VR) technology on sport spectators’ flow experience and satisfaction. Comput Hum Behav. 2019;93:346–56.

[97] Nguyen TBT, Le TBN, Chau NT. How VR technological features prompt tourists’ visiting intention: An integrated approach. Sustainability. 2023;15(6).

[98] Novak TP, Hoffman DL, Yung Y-F. Measuring the Customer Experience in Online Environments: A Structural Modeling Approach. Marketing Science. 2000;19(1):22–42. doi: 10.1287/mksc.19.1.22.15184

[99] Chi H-K, Huang K-C, Nguyen HM. Elements of destination brand equity and destination familiarity regarding travel intention. Journal of Retailing and Consumer Services. 2020;52:101728. doi: 10.1016/j.jretconser.2018.12.012

[100] Gu Y, Cheng XS, Shen J. Design shopping as an experience: Exploring the effect of the live-streaming shopping characteristics on consumers’ participation intention and memorable experience. Inf Manage. 2023;60(5).

[101] Jianguo G. A study on the influence of museum smart tourism technology experience on tourists’ subjective well-being and willingness to revisit. 2022.

[102] Oliver MB, Raney AA. Entertainment as Pleasurable and Meaningful: Identifying Hedonic and Eudaimonic Motivations for Entertainment Consumption. Journal of Communication. 2011;61(5):984–1004. doi: 10.1111/j.1460-2466.2011.01585.x

[103] Yuan YH, Wu CK. Relationships among experiential marketing, experiential value, and customer satisfaction. Journal of Hospitality & Tourism Research. 2008;32(3):387–410.

[104] Verhagen T, Feldberg F, van den Hooff B, Meents S, Merikivi J. Satisfaction with virtual worlds: an integrated model of experiential value. Inf Manage. 2011;48(6):201–7.

[105] van der Heijden H. Factors influencing the usage of websites: the case of a generic portal in The Netherlands. Information & Management. 2003;40(6):541–9. doi: 10.1016/s0378-7206(02)00079-4

[106] Vishwakarma P, Mukherjee S, Datta B. Travelers’ intention to adopt virtual reality: A consumer value perspective. Journal of Destination Marketing & Management. 2020;17:100456. doi: 10.1016/j.jdmm.2020.100456

[107] Leveau P, Camus et S. Embodiment, immersion, and enjoyment in virtual reality marketing experiences. Psychology and Marketing. 2023;40(7):1329–43. doi: 10.1002/mar.21822

[108] Wojciechowski R, Cellary W. Evaluation of learners’ attitude toward learning in ARIES augmented reality environments. Computers & Education. 2013;68:570–85. doi: 10.1016/j.compedu.2013.02.014

[109] Chung N, Han H, Joun Y. Tourists’ intention to visit a destination: The role of augmented reality (AR) application for a heritage site. Comput Hum Behav. 2015;50:588–99.

[110] Jeong M, Shin HH. Tourists’ Experiences with Smart Tourism Technology at Smart Destinations and Their Behavior Intentions. Journal of Travel Research. 2019;59(8):1464–77. doi: 10.1177/0047287519883034

[111] Tan W-K. The relationship between smartphone usage, tourist experience and trip satisfaction in the context of a nature-based destination. Telematics and Informatics. 2017;34(2):614–27. doi: 10.1016/j.tele.2016.10.004

[112] Pietschmann D, Valtin G, Ohler P. The Effect of Authentic Input Devices on Computer Game Immersion. In: Unger JFaA. In Computer Games and New Media Cultures: A Handbook of Digital Games Studies. 2012. p. 279–92.

[113] Hosany S, Witham M. Dimensions of Cruisers’ Experiences, Satisfaction, and Intention to Recommend. Journal of Travel Research. 2009;49(3):351–64. doi: 10.1177/0047287509346859

[114] Quadri-Felitti D, Fiore AM. Experience economy constructs as a framework for understanding wine tourism. Journal of Vacation Marketing. 2012;18(1):3–15. doi: 10.1177/1356766711432222

[115] Han S-L, Kim J, An M. The Role of VR Shopping in Digitalization of SCM for Sustainable Management: Application of SOR Model and Experience Economy. Sustainability. 2023;15(2):1277. doi: 10.3390/su15021277

[116] Kim K, Hwang J, Zo H, Lee H. Understanding users’ continuance intention toward smartphone augmented reality applications. Information Development. 2014;32(2):161–74. doi: 10.1177/0266666914535119

[117] Rese A, Baier D, Geyer-Schulz A, Schreiber S. How augmented reality apps are accepted by consumers: A comparative analysis using scales and opinions. Technol Forecast Soc Chang. 2017;124:306–19.

[118] Hair JF, Sarstedt M, Ringle CM, Mena JA. An assessment of the use of partial least squares structural equation modeling in marketing research. J of the Acad Mark Sci. 2011;40(3):414–33. doi: 10.1007/s11747-011-0261-6

[119] Hair JF Jr, Hult GTM, Ringle C, Sarstedt M. A primer on partial least squares structural equation modeling (PLS-SEM) Sage Publications. Thousand Oaks, CA, USA. 2016.

[120] Hair J, Anderson R, Tatham R, Black W. Multivariate data analysis. 5th ed. Englewood Cliffs, NJ: Prentice-Hall. 1998.

[121] Podsakoff PM, MacKenzie SB, Lee J-Y, Podsakoff NP. Common method biases in behavioral research: a critical review of the literature and recommended remedies. J Appl Psychol. 2003;88(5):879–903. doi: 10.1037/0021-9010.88.5.879 14516251

[122] Fornell C, Larcker DF. Evaluating Structural Equation Models with Unobservable Variables and Measurement Error. Journal of Marketing Research. 1981;18(1):39. doi: 10.2307/3151312

[123] Nunnally J. Psychometric theory 3E. New York, NY: Tata McGraw-hill education. 1994.

[124] Falk RF, Miller NB. A primer for soft modeling. Akron: University of Akron Press. 1992.

[125] Tenenhaus M, Vinzi VE, Chatelin Y-M, Lauro C. PLS path modeling. Computational Statistics & Data Analysis. 2005;48(1):159–205. doi: 10.1016/j.csda.2004.03.005

[126] Hair JF, Ringle CM, Sarstedt M. PLS-SEM: Indeed a Silver Bullet. Journal of Marketing Theory and Practice. 2011;19(2):139–52. doi: 10.2753/mtp1069-6679190202

[127] Ch’ng E, Cai SD, Leow FT, Zhang TE. Adoption and use of emerging cultural technologies in China’s museums. J Cult Herit. 2019;37:170–80.

[128] Jiang Y, Guo RL, Ma FF. Interactive multimedia system for Chinese traditional costumes. In: Bournemouth, UK, 2017.

[129] Hein HS. The museum in transition: A philosophical perspective. Smithsonian Institution. 2014.

[130] Poushneh A, Vasquez-Parraga AZ. Discernible impact of augmented reality on retail customer’s experience, satisfaction and willingness to buy. Journal of Retailing and Consumer Services. 2017;34:229–34. doi: 10.1016/j.jretconser.2016.10.005

[131] Jiménez-Barreto J, Rubio N, Campo S. Destination brand authenticity: What an experiential simulacrum! A multigroup analysis of its antecedents and outcomes through official online platforms. Tourism Management. 2020;77:104022. doi: 10.1016/j.tourman.2019.104022

[132] Yi X, Fu X, Yu L, Jiang L. Authenticity and loyalty at heritage sites: The moderation effect of postmodern authenticity. Tourism Management. 2018;67:411–24. doi: 10.1016/j.tourman.2018.01.013

[133] Huang J, Zhou L. Timing of web personalization in mobile shopping: a perspective from uses and gratifications theory. Comput Hum Behav. 2018;88:103–13.

[134] Verhagen T, van Dolen W. The influence of online store beliefs on consumer online impulse buying: A model and empirical application. Inf Manage. 2011;48(8):320–7.

[135] Lee H, Chung N, Jung T. Examining the Cultural Differences in Acceptance of Mobile Augmented Reality: Comparison of South Korea and Ireland. Springer International Publishing. 2015.

[136] Oh H, Fiore AM, Jeong M. Measuring experience economy concepts: tourism applications. J Travel Res. 2023;62(7):1619-.

[137] Flavián C, Ibáñez-Sánchez S, Orús C. The impact of virtual, augmented and mixed reality technologies on the customer experience. Journal of Business Research. 2019;100:547–60. doi: 10.1016/j.jbusres.2018.10.050

[138] Coyle JR, Thorson E. The effects of progressive levels of interactivity and vividness in web marketing sites. Journal of Advertising. 2001;30(3):65–77.

[139] Steuer J. Defining virtual reality - dimensions determining telepresence. Journal of Communication. 1992;42(4):73–93.

[140] Nechita F, Rezeanu C-I. Augmenting Museum Communication Services to Create Young Audiences. Sustainability. 2019;11(20):5830. doi: 10.3390/su11205830

[141] Peng LF, Hassan Z, Basit A. Store Attributes: A Sustainable Strategy to Influence Customer Satisfaction and Purchase Intention. ijmbe. 2018;1(1):19. doi: 10.32455/ijmbe.v1i1.51

[142] Nian SF, Zhang HL, Mao L, Zhao WJ, Zhang H, Lu YH. How outstanding universal value, service quality and place attachment influences tourist intention towards world heritage conservation: A case study of Mount Sanqingshan National Park, China. Sustainability. 2019;11(12).

