# Supplementary material for: Factors influencing visitors’ use of augmented reality technology in museum guided tours
Source: PLoS One. 2025 Oct 7;20(10):e0332688. doi: 10.1371/journal.pone.0332688 (PMC12503337; doi:10.1371/journal.pone.0332688)
Supplement: S1 Appendix — (DOC) [file pone.0332688.s001.doc]

**Appendix A Questionnaire scale**

| **Variable** | **Items** |
| --- | --- |
| Authenticity | The museum AR function provides accurate information. |
|  | Using the museum AR function provided me with authentic experiences. |
|  | Using the museum AR function provided me with genuine experiences. |
| Individuation | The museum AR function provide recommendations to suit my preferences. |
|  | The museum AR function recommend activities, dining and other services based on my usage information. |
|  | The museum AR function provide dialect navigation, star voice navigation and other special services. |
|  | The museum AR function provide me with personalized tour customization service. |
| Aesthetics | The museum AR function made me feel alive. |
|  | The museum AR function interface design is well coordinated. |
|  | The museum AR function design is attractive to me. |
| Interactivity | The museum AR function interface design is friendly. |
|  | I can move freely and get information in the museum AR environment. |
|  | The museum AR function has great interactive feedback. |
| Satisfaction | I don't complain when using the museum AR function. |
|  | The museum AR function fulfilled my requirements. |
|  | The experience of using the museum AR function was satisfactory overall. |
| Perceived Enjoyment | I never get bored when using the museum AR function. |
|  | I get lots of fun when using the museum AR function. |
|  | I am interested in the museum AR function. |
| Intention to Use | When visiting museums, I will use the museum AR function. |
|  | I recommend museum AR to my relatives and friends. |
|  | My willingness to visit a monument with AR function is higher than to visit those not having it. |
